# Supplementary material for: Home Range and Habitat Use of Breeding Black-necked Cranes
Source: Animals (Basel). 2020 Oct 28;10(11):1975. doi: 10.3390/ani10111975 (PMC7694124; doi:10.3390/ani10111975)
Supplement: Supplementary file 1 [file animals-10-01975-s001.pdf]

Environmental characteristics of the three habitats

We investigated the differences of vegetation height and water depth among marsh, marsh meadow, and meadow in 2015. We chose four Sample plot (about 1 ha /each plot) in each habitat. We designed separately ten sample quadrats in each sample plot in May, July and September, 2015. Consequently, we collected 360 sample quadrats data. Each quadrat was 2*2 m^2. We measured vegetation height by a direct measurement method. We placed a hand lightly on the vegetation at the level below which about 80% of the vegetation is estimated by eye to be growing (thus ignoring occasional tall stalks), then reading this height on a ruler (Hodgson et al. 1971; Stewart et al. 2001). The water depth was the height of silt surface to water surface.

Vegetation height of marsh was 40.7 ± 2.5 cm, water depth of marsh was 35.3 ± 0.9 cm (n=120, Mean ± SD). Vegetation height of marsh meadow was 10.2 ± 0.5 cm, water depth of marsh meadow was 7.2 ± 0.5 cm. Vegetation height of meadow was 5.6 ± 0.3 cm, water depth of meadow was 0.8 ± 0.2 cm. There were significant difference of vegetation height compared among marsh, marsh meadow, and meadow (GLM: $X^2 = 79.772$, $P < 0.001$), there were significant difference of water depth compared among marsh, marsh meadow, and meadow (GLM: $X^2 = 222.381$, $P < 0.001$).

Reference

Hodgson J, Tayler JC, Lonsdale CR (1971). The relationship between intensity of grazing and the herbage consumption and growth of calves. *Grass and Forage Science* **26**, 231-38.

Stewart KEJ, Bourn NAD, Thomas JA (2001). An evaluation of three quick methods commonly used to assess sward height in ecology. *The Journal of applied ecology* **38**, 1148-54.
