# Supplementary material for: Home Range and Habitat Use of Breeding Black-necked Cranes
Source: Animals (Basel). 2020 Oct 28;10(11):1975. doi: 10.3390/ani10111975 (PMC7694124; doi:10.3390/ani10111975)
Supplement: Supplementary file 2 [file animals-10-01975-s002.pdf]

The home range size of the Black-necked Crane pair and habitat patch size (ha, mean ± SE) within the home range during the four breeding stages.

Method	Type	P-I (n = 23)	IN (n = 16)	P-F (n = 15)	F-F (n = 13)
50%FKP	HRs	19.98 ± 4.94	27.158 ± 8.75	14.59 ± 2.97	19.13 ± 3.39
	Marsh	2.82 ± 0.65	2.79 ± 0.67	2.37 ± 0.80	1.35 ± 0.79
	Marsh meadow	5.08 ± 1.45	7.28 ± 2.59	3.44 ± 1.08	3.03 ± 0.98
	Meadow	12.05 ± 3.79	17.11 ± 6.75	8.79 ± 2.51	14.75 ± 3.59
95%FKP	HRs	141.68 ± 35.30	151.87 ± 38.30	92.11 ± 17.25	111.48 ± 18.34
	Marsh	19.63 ± 3.13	19.84 ± 4.08	14.63 ± 3.52	9.86 ± 4.47
	Marsh meadow	36.15 ± 8.67	37.18 ± 11.20	26.89 ± 8.05	21.26 ± 4.84
	Meadow	84.86 ± 26.99	94.71 ± 29.48	50.59 ± 12.45	80.36 ± 19.06

FKP denotes the fixed-kernel polygons method, HRs denotes the home range size; P-I denotes the pre-incubation stage, IN denotes the incubation stage, P-F denotes the post-fledging stage, and F-F denotes the fully-fledged stage.
